# Changing Rhizosphere Microbial Community and Metabolites with Developmental Stages of *Coleus barbatus*

**DOI:** 10.3390/microorganisms11030705

**Published:** 2023-03-09

**Authors:** Vijay Lakshmi Jamwal, Irshad Ahmad Rather, Sajad Ahmed, Amit Kumar, Sumit G. Gandhi

**Affiliations:** 1 CSIR-Indian Institute of Integrative Medicine, Jammu 180001, India; 2 Academy of Scientific and Innovative Research (AcSIR), Ghaziabad 201002, India; 3 Department of Botanical and Environmental Sciences, Guru Nanak Dev University, Amritsar 143005, India

**Keywords:** *Coleus forskohlii*, forskolin, medicinal plants, metagenomics, rhizobacteria, secondary metabolites

## Abstract

*Coleus barbatus* is a medicinal herb belonging to Lamiaceae. It is the only living organism known to produce forskolin, which is a labdane diterpene and is reported to activate adenylate cyclase. Microbes associated with plants play an important role in maintaining plant health. Recently, the targeted application of beneficial plant-associated microbes and their combinations in abiotic and biotic stress tolerance has gained momentum. In this work, we carried out the rhizosphere metagenome sequencing of *C. barbatus* at different developmental stages to understand how rhizosphere microflora are affected by and affect the metabolite content in plants. We found that the *Kaistobacter* genus was abundantly present in the rhizosphere of *C. barbatus* and its accumulation pattern appears to correlate with the quantities of forskolin in the roots at different developmental stages. Members of the *Phoma* genus, known for several pathogenic species, were in lower numbers in the *C. barbatus* rhizosphere in comparison with *C. blumei*. To our knowledge, this is the first metagenomic study of the rhizospheric microbiome of *C. barbatus*, which may help to explore and exploit the culturable and non-culturable microbial diversity present in the rhizosphere.

## 1. Introduction

Plants growing in a particular soil affect the physical, biological, and chemical properties of the rhizosphere in the vicinity of the roots. The deposition of nutrients by roots into the rhizosphere leads to the enrichment of microorganisms because root exudates support the increased microbial growth in soil, and this phenomenon is called the “rhizosphere effect” [1,2]. Through root exudation, plants attract several beneficial microbes such as arbuscular mycorrhizal fungi and nitrogen-fixing bacteria, but the selection mechanism of plants for the beneficial response to avoid harmful microbial candidates is not known [3]. In recent decades, researchers all over the world have started to intensively examine the dynamics of community and structure as well as the functions of bacterial and fungal communities associated with plant roots [4,5,6,7]. The magnitude of the diversity of the rhizospheric microbial community changes from thousands to millions with the age and developmental stages of the plant [8,9,10,11,12,13]. Dibner et al. (2021) argued that time and microbial succession more strongly affect microbial assemblage than the plant developmental stage [14]. Regarding the impact of disturbance on existing indigenous soil microbial communities by introducing plants, several studies demonstrated that the soil type has a major effect on the structure of microbial communities [15,16], while others have the opposite view, as the plants play a major role in the community structure [17,18]. The diversity in the rhizosphere microbial community is also influenced by several host-related factors [14,19,20], such as host plant species, genotype of plant, richness of plant community, interactions among microbes that span from facultative to antagonistic, traits of the plant-like growth rate, and root length [21,22,23,24,25]. The change in microbial diversity via the plant developmental stages occurs due to the changes in the pattern of root exudation that affect nutrient enrichment for the microbes or changes in root architecture that affect the physical habitat available for microbes [26,27].

Rhizospheric microbial diversity has been investigated in the model plant *Arabidopsis thaliana*, as well as in several crop species such as *Pisum sativum* (pea), *Solanum tuberosum* (potato), and *Triticum aestivum* (wheat), where changes have been shown in the bacterial and fungal diversity associated with the developmental stages of the plant. However, in *Beta vulgaris* (sugar beet) no such correlation has been observed [9,28]. This evidence indicates that the developmental stage of a particular plant could be important for the determination of rhizosphere microbial diversity. Furthermore, similar detailed studies assessing the impact of microbial associations on plant physiology and viceversa are relatively scarce in plant systems [9,29,30]. However, the effects of the developmental stages of *Coleus barbatus* (Andrews) Benth. ex G.Don (Lamiaceae) on the microbial communities present in the rhizosphere have not been investigated until now.

The perennial herb *C. barbatus* is a valuable medicinal plant cultivated in subtropical, warm, temperate areas of India, Thailand, and Myanmar. The roots of this plant store compounds that have been used to treat various diseases related to the heart, lungs, hypothyroidism, and several other functions. The major biologically active ingredient of *C. barbatus* root extract is forskolin, which accumulates in the roots [31]. Forskolin is a potent activator of adenylate cyclase, which increases intracellular cyclic 3′, 5′-adenosine monophosphate (cAMP) levels in different mammalian membranes and intact tissues [32,33,34,35,36,37,38]. This intrinsic property of forskolin explains the use of the compound as a potential therapeutic agent for diseases such as hypertension, glaucoma, asthma, and obesity [31,39,40]. *Coleus blumei* Benth. (Lamiaceae) is an evergreen perennial ornamental plant. *C. blumei* is known for the production of an active polyphenol compound, rosmarinic acid, which has antioxidant, anti-inflammatory, and antiviral activities [41,42]. Although this species belongs to the genus *Coleus* and grows in similar environmental conditions as *C. barbatus*, it is not known to produce or accumulate forskolin.

Therefore, the significant change in microbial association in the rhizosphere during plant development and growth needs more attention. The present study, where we attempted to analyze the rhizosphere microbiome of *C. barbatus* is the first of its kind. Thus, the study of microbes associated with *C. barbatus* would not only allow exploring and exploiting the untapped microbial diversity but also provide some understanding of the impact of plant metabolites on the rhizosphere microflora and vice versa.

## 2. Materials and Methods

### 2.1. Collection of Samples

*C. barbatus* (Syn. *Coleus forskohlii*) and *C. blumei* cuttings were raised in experimental plots at CSIR-IIIM (Jammu) during the month of December. Rhizosphere soil samples were collected at different developmental stages of *C. barbatus* and *C. blumei*. Plants were uprooted and the soil attached to the roots was collected as rhizosphere soil [43,44]. Soil collected at 0 d, i.e., at the time of planting the cuttings, was taken as control soil (Control). Rhizosphere soil of *C. barbatus* was collected on 45 d (Cf45d), 90 d (Cf90d), and 180 d (Cf180d) of the plantation. Rhizosphere soil of *C. blumei* at 180 d of plantation (Cb180d) was also collected for comparison. Roots of *C. barbatus* were also collected at 0, 45, 90, and 180 d. Samples from three different plants were collected for each time point and the samples were pooled to form a composite sample for each time point. For 0 d, the terminal portion of the stem cuttings were collected as control.

### 2.2. DNA Isolation from Soil Samples

Rhizosphere soil DNA was isolated from 250–500 mg of a sample using a HiPurA^®^ Soil DNA Purification Kit (HiMedia, Mumbai, India), as per manufacturer’s instructions. The quality and quantity of isolated rhizosphere soil DNA were assessed on 1% agarose gel and a spectrophotometer (Nanodrop 8000, Thermo Fisher Scientific, Waltham, MA, USA), respectively. The quantification of isolated DNA was also carried out through the Qubit dsDNA HS Assay kit (Life Technologies, Carlsbad, CA, USA) using Qubit^®^ 2.0 Fluorometer (Life Technologies, Carlsbad, CA, USA).

### 2.3. Preparation of Libraries

The amplicon libraries were prepared using Nextera XT Index Kit (Illumina Inc., San Diego, CA, USA) as per the Metagenomic Sequencing Library preparation protocol. The primers used for the amplification of the 16S rDNA gene and ITS (Internal Transcribed Spacer) regions are mentioned in Table 1. The DNA was fragmented and tagged with the Illumina adapters. The tagmented DNA was amplified using Index 1 and Index 2 primers for cluster generation according to the standard Illumina protocol. The amplicon libraries were purified using 1X AMpureXP beads. The amplicons were checked and quantified on an Agilent 2100 Bioanalyzer (Agilent Technologies, Santa Clara, CA, USA) and a fluorometer using a Qubit dsDNA HS Assay kit (Life Technologies, Carlsbad, CA, USA), respectively.

### 2.4. Cluster Generation and Sequencing

The next-generation sequencing (NGS) for Control-16S, Cb180d-16S, Cf180d-16S, Cf90d-16S, and Cf45d-16S, as well as Control-ITS, Cb180d-ITS, Cf180d-ITS, Cf90d-ITS, and Cf45d-ITS was performed on the Illumina MiSeq platform. The concentration of DNA in the libraries was quantified using a Qubit fluorometer (Life Technologies, Carlsbad, CA, USA). The mean peak sizes of the amplicons in the library were assessed using a bioanalyzer. A concentration of the libraries of 10–20 pM was loaded onto MiSeq for cluster generation and sequencing. The template fragments were allowed to sequence on both sides through paired-end sequencing. The kit reagents were used in binding samples to complementarily adapter oligos on the paired-end flow cell. The adapters were designed in a manner so that, during sequencing, the adapters allowed selective cleavage of the forward strands after re-synthesis of the reverse strand. The opposite end of the fragment was sequenced from the copied reverse.

### 2.5. Computational Analysis

16S and ITS metagenome analysis were performed using Quantitative Insights Into Microbial Ecology (QIIME) [45].

#### 2.5.1. QIIME Analysis

QIIME is comprehensive software comprising tools and algorithms such as FastTree for phylogeny inference based on maximum-likelihood [46]. The RDP (Ribosomal Database Project) classifier (for bacterial 16S) and the UNITE database (for fungal ITS) were used for the assignment of taxonomic data [47,48,49,50,51].

The raw data were processed by stitching the paired-end data into single end reads. The analysis was divided into two stages: upstream and downstream. In upstream analysis, raw data were analyzed. Based on the sequence similarities, all the sequences from all the samples were clustered into Operational Taxonomic Units (OTUs). The clustering of OTU was performed using UCLUST, which is frequently used to represent some degree of taxonomic relatedness [52]. Each resulting cluster typically represents a genus. The representative sequence was picked up from each OTU for taxonomic identification. These representative sequences were analyzed by adding them to existing high-quality template alignments, using Python Nearest Alignment Space Termination (PyNAST) [45,53]. Taxonomic assignment to microbial lineages was carried out after alignment.

#### 2.5.2. Diversity Comparison

The Shannon alpha diversity index was used as diversity metric to compare the types of communities. The beta diversity index was also used as a diversity metric. Species abundance was plotted using rank abundance plot.

### 2.6. Metabolite Quantification

#### 2.6.1. Preparation of Extracts for Phytochemical Quantification

Crude extracts were prepared from different tissues of *C. barbatus* at different developmental stages. Shade-dried plant tissues (2 g) were powdered and extracted thrice with 10 mL of methanol at 30 °C (with sonication) for 3 h. Methanolic extracts were used for quantification of total phenolics, flavonoids, and alkaloids.

#### 2.6.2. Flavonoid Quantification

Total flavonoids in different tissues (leaves, stems, and roots) were quantified using spectrophotometry as previously described [54].

#### 2.6.3. Phenolic Quantification

Total phenolic content in different tissues of *C. barbatus* was measured using the Folin–Ciocalteu reagent method as described by Pinelo et al. (2004) [55]. The phenolic content was expressed as mg of gallic acid equivalent (GAE) per gram of dry weight of the sample. The experiment was done in triplicate.

#### 2.6.4. Terpenoid Quantification

Total terpenoid content in different tissues of *C. barbatus* was quantified by adopting the protocol of Ghorai et al. (2012) [56]. Briefly, 3.5 mL of ice-cold 95% (*v*/*v*) methanol was added to 500 mg of fresh tissue, followed by homogenization in an ice-cold mortar and pestle. The samples were incubated at room temperature for 2 days in the dark. After incubation samples were centrifuged (4000× *g*, 15 min) and the supernatant was collected, to 1.5 mL of chloroform in a 2 mL tube, 200 µL of sample supernatant was added. The mixture was vortexed and then incubated at room temperature for 3 min. A quantity of 100 µL of sulfuric acid was added to each tube followed by incubation at room temperature in the dark for 2 h. After incubation, a reddish-brown precipitate was formed. Supernatant was discarded without disturbing the precipitate. Precipitates were dissolved in 1.5 mL of 95% (*v*/*v*) methanol. Absorbance was read at 538 nm with their respective controls. Linalool was used as standard (1.29–12.9 µM, R = 0.996).

#### 2.6.5. Alkaloid Quantification

Total alkaloid content was measured in leaves, stems, and roots of *C. barbatus* using bromocresol green solution (BCG) [57]. Extract was dissolved in 2 N HCl and then filtered. A quantity of 1 mL of this solution was extracted thrice with 10 mL of chloroform. A quantity of 0.1 N NaOH was used to neutralize the pH of the extracted solution. A quantity of 10 mL of a mixture containing BCG and phosphate buffer (1:1) was added to the extracted solution. The mixture was shaken vigorously and extracted using chloroform. Subsequently, the extract was diluted to 10 mL using chloroform. Finally, absorbance was read at 470 nm, with their respective controls and blank. Atropine was used as standard (10–100 µg, R = 0.994).

#### 2.6.6. Forskolin Quantification

The roots of *C. barbatus* collected at 0, 45, 90, and 180 d were used for preparing crude extracts. The dried roots were powdered and 100 mg of root powder was extracted thrice with acetonitrile at 30 °C (with sonication) for 2 h. The obtained crude extracts were concentrated and dissolved in acetonitrile. The extracts were filtered through a 0.20 µm filter (Millipore, Billerica, MA, USA) and subjected to high-performance liquid chromatography (HPLC) analysis for quantification of forskolin. Pure forskolin (Sigma-Aldrich, St. Louis, MO, USA) standard was dissolved in MS-grade acetonitrile to obtain a stock concentration of 1 mg/mL. The analysis was carried out using an HPLC system (Shimadzu CLASS-VP V 6.14 SPI model, Kyoto, Japan) for the detection of forskolin in the samples. Acetonitrile:formic acid (99.5:0.5; *v*/*v*) was used as mobile phase for performing the analysis. A quantity of 10 μL of injection volume of the samples was used for analysis. The detection was undertaken on the basis of retention time of reference standard (forskolin).

## 3. Results

### 3.1. Quantification of Bulk Phytochemicals and Forskolin at Different Developmental Stages

**Figure 1:**
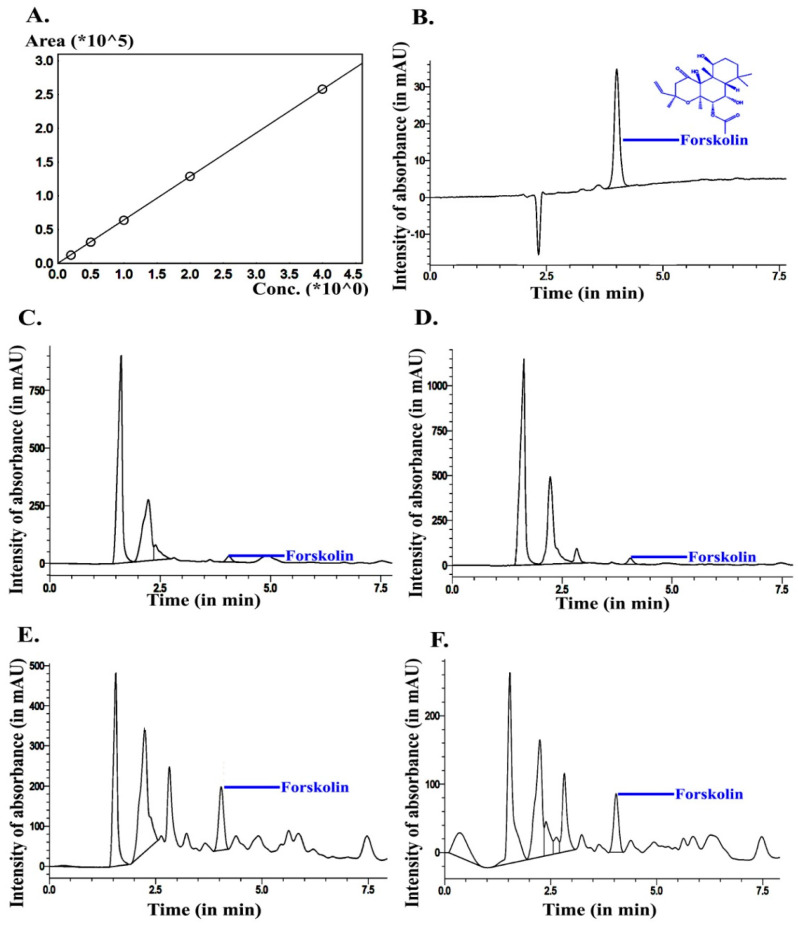
Forskolin quantification. (**A**) Standard curve; (**B**) chromatogram of standard; (**C**) *C. barbatus* root extract at 0 d; (**D**) *C. barbatus* root extract 45 d; (**E**) *C. barbatus* root extract 90 d; (**F**) *C. barbatus* root extract 180 d.

**Figure 2:**
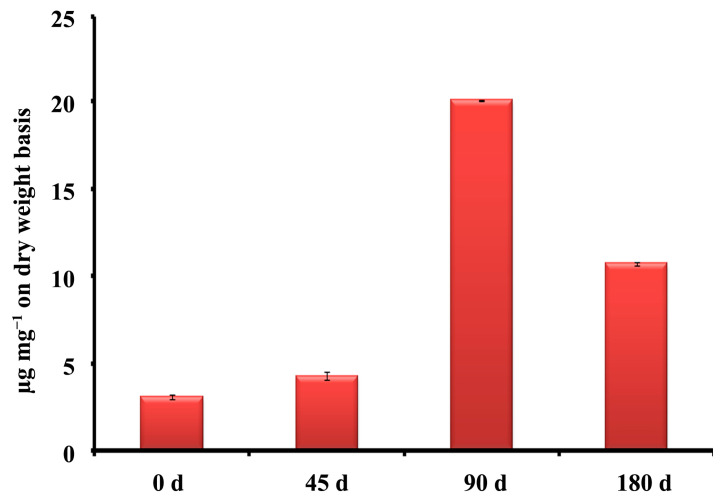
Forskolin content in root extract of 0, 45, 90, and 180 d old *C. barbatus* plant.

**Figure 3:**
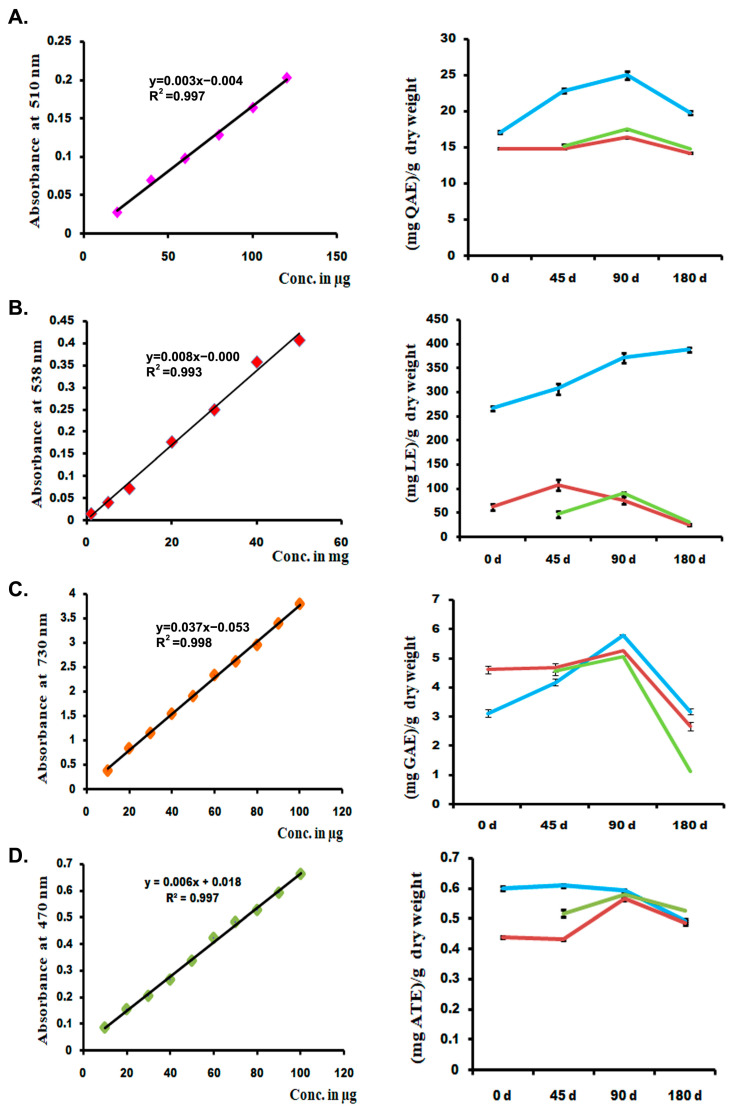
Metabolite quantification: (**A**) total flavonoid; (**B**) total terpenoid; (**C**) total phenolics; (**D**) total alkaloid.

Generally, it was observed after 30–45 d of plantation of *C. barbatus* that the development of roots started, and at 90 d the roots were developed completely and may have maximum forskolin content. Accordingly, to understand the effect of forskolin or other related metabolites accumulated in the roots of *C. barbatus* and the impact of different developmental stages on rhizosphere microflora, we sampled plants at 45 d (Cf45d), 90 d (Cf90d), and 180 d (Cf180d) after planting for study. Profiling of bulk phytochemicals and the key metabolite forskolin in different tissues during the development of *C. barbatus* showed that the 90 d old plant was metabolically more active. The HPLC analysis of crude extracts showed that forskolin content reached a maximum in 90 d old *C. barbatus* root extract, followed by 180 d old *C. barbatus* root extract, and was the lowest in 0 d and 45 d *C. barbatus* root extract (Figure 1, Figure 2 and Figure 3).

### 3.2. Sequencing of Rhizospheric DNA

The read statistics of data generated are shown in Table 2. We observed that the alpha diversity of all samples was less than that of the control sample (Table 3). However, when the bacterial diversity was compared, we found that the rhizosphere of *C. barbatus* showed richness in bacterial diversity as compared to the rhizosphere of *C. blumei*, whereas fungal diversity showed the opposite trends.

### 3.3. Taxonomic Composition of Rhizosphere

#### 3.3.1. Bacterial Diversity

**Figure 4:**
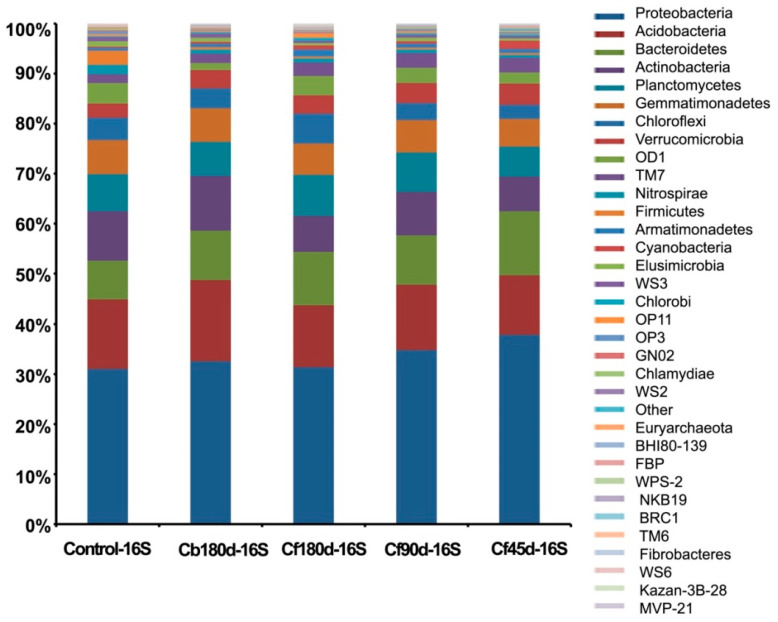
Bacterial diversity in rhizosphere soil at different developmental stages of *C. barbatus* at phylum level.

**Figure 5:**
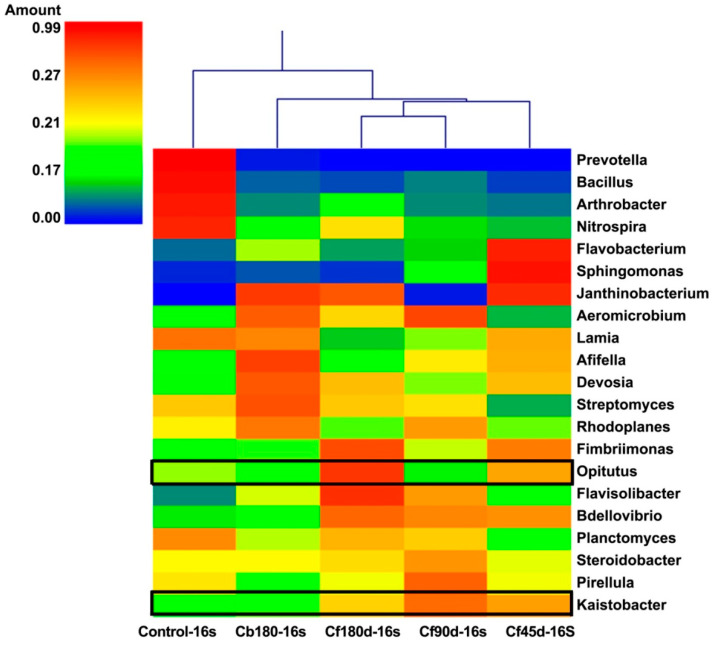
Heatmap showing abundance of bacterial diversity in rhizosphere soil at different developmental stages of *C. barbatus* at genus level.

In the present study, Proteobacteria, Acidobacteria, Actinobacteria, Bacteroidetes, and Planctomycetes were the most abundant phyla detected in the 16S metagenomic library. The highest percentage of Phylum Proteobacteria hits was detected in Cf45d-16S (37.8%), followed by Cf90d-16S (34.7%), as compared to the Control-16S (30.9%). Phylum Acidobacteria were abundant in Cb180d-16S (16.2%),but less abundant in Cf90d-16S (13%), Cf180d-16S (12.4%), and Cf45d-16S (11.8%) as compared to the Control-16S (13.8%). Phylum Actinobacteria had same pattern of abundance as Acidobacteria, i.e., highest in Cb180d-16S (11%) followed by Control-16S (9.8%) and less in Cf90d-16S (8.7%), Cf180d-16S (7.2%), and Cf45d-16S (7%). Phylum Bacteroidetes was abundant in Cf45d-16S (12.80%) followed by Cf180d-16S (10.60%). Phylum Chloroflexi was dominantly detected in Cf180d-16S (5.90%) (Figure 4). At class level, Alphaproteobacteria, Acidobacteria-6, Betaproteobacteria, Deltaproteobacteria, Gammaproteobacteria, and Phycisphaerae were the classes with maximum hits (Supplementary Figure S1). Sphingomonadales, Acidimicrobiales, Xanthomonadales, and Rhizobiales were the abundant orders detected in 16S metagenomic data of all samples (Supplementary Figure S2). At family level, Sphingomonadaceae, Chitinophagaceae, Cytophagaceae, and Hyphomicrobiaceae were the dominant bacterial families detected (Supplementary Figure S3). On analyzing data at genus level, the *Kaistobacter* genus was found to be most abundant in all samples, followed by *Bacillus* in Control-16S, *Rhodoplanes* in Cb180d-16S and Cf90d-16S, *Flavisolibacter* in Cf180d-16S, and *Sphingomonas* in the Cf45d-16S sample (Figure 5).

#### 3.3.2. Fungal Diversity

**Figure 6:**
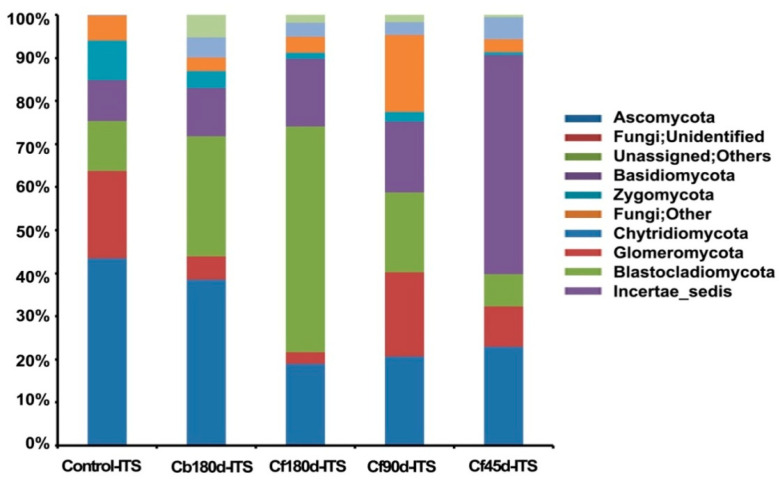
Fungal diversity in rhizosphere soil at different developmental stages of *C. barbatus* at phylum level.

**Figure 7:**
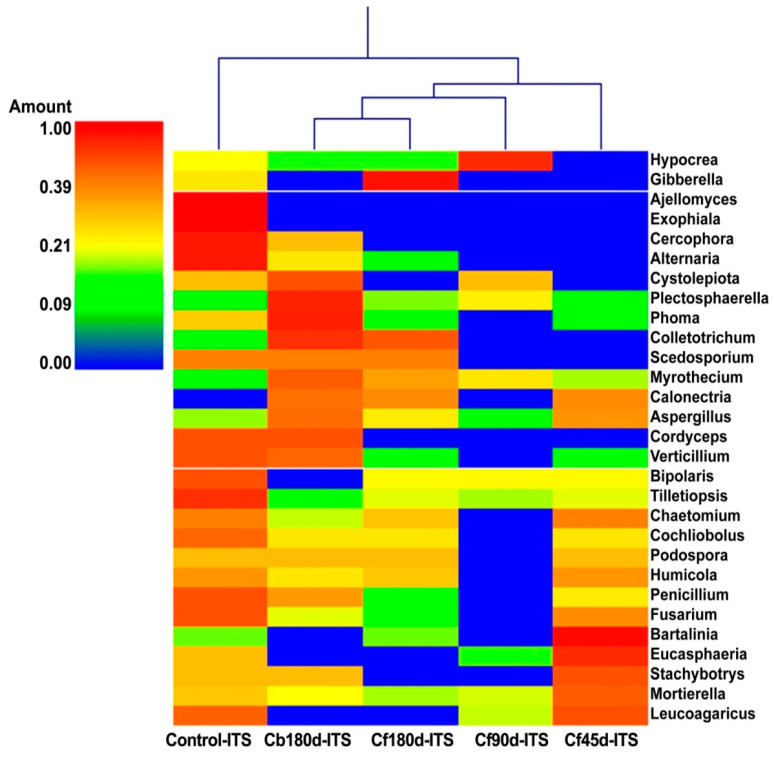
Heatmap showing abundance of fungal diversity in rhizosphere soil at different developmental stages of *C. barbatus* at genus level.

Ascomycota was the most abundant phylum detected in ITS rhizospheric metagenomic data of all samples. The percentage abundance of Phylum Ascomycota was highest in the control (43.50%) sample, followed by Cb180d-ITS (38.50%), and was lower in Cf180d-ITS (19.00%), Cf90d-ITS (20.70%), and Cf45d-ITS (22.90%) (Figure 6). The presence of Agaricomycetes, Sordariomycetes, Dothideomycetes, Eurotiomycetes, Exobasidiomycetes, and Chytridiomycetes classes was dominant in all samples. The ITS metagenomic data of Cf90d-ITS detected maximum hits for class Agaricomycetes, i.e., 18.90%, followed by control-ITS (17.70%), Cf45d-ITS (8.60%), Cf180d-ITS (1.00%), and Cb180d-ITS (0.80%). Class Sordariomycetes was most abundantly present in Cf45d-ITS (21.60%), followed by Cb180d-ITS (20.80%), and less abundantly present in Cf90d-ITS (12.20%) and Cf180d-ITS (12.00%) when compared with control (13.30%). Class Dothideomycetes was dominant in Cb180d-ITS (11.00%) when compared with control-ITS (8.70%) and other samples: Cf180d-ITS (1.50%), Cf90d-ITS (1.40%), and Cf45d-ITS (0.10%) (Supplementary Figure S4). Agaricales, Mortierellales, and Hypocreales were the most dominant orders present in all samples (Supplementary Figure S5). Agaricaceae, Chaetomiaceae, Pleosporaceae, Mortierellaceae, and Nectriaceae were the families abundantly present in all samples (Supplementary Figure S6). At the genus level, the *Leucoagaricus* genus was found to be most abundant in Control-ITS, Cf90d-ITS, and Cf45d-ITS samples, whereas the Cf180d-ITS sample was enriched with *Mortierella*. The *Phoma* genus was abundant in Cb180d-ITS as compared to the control, and rarely present in Cf180d-ITS, Cf90d-ITS, or Cf45d-ITS (Figure 7).

**Figure 8:**
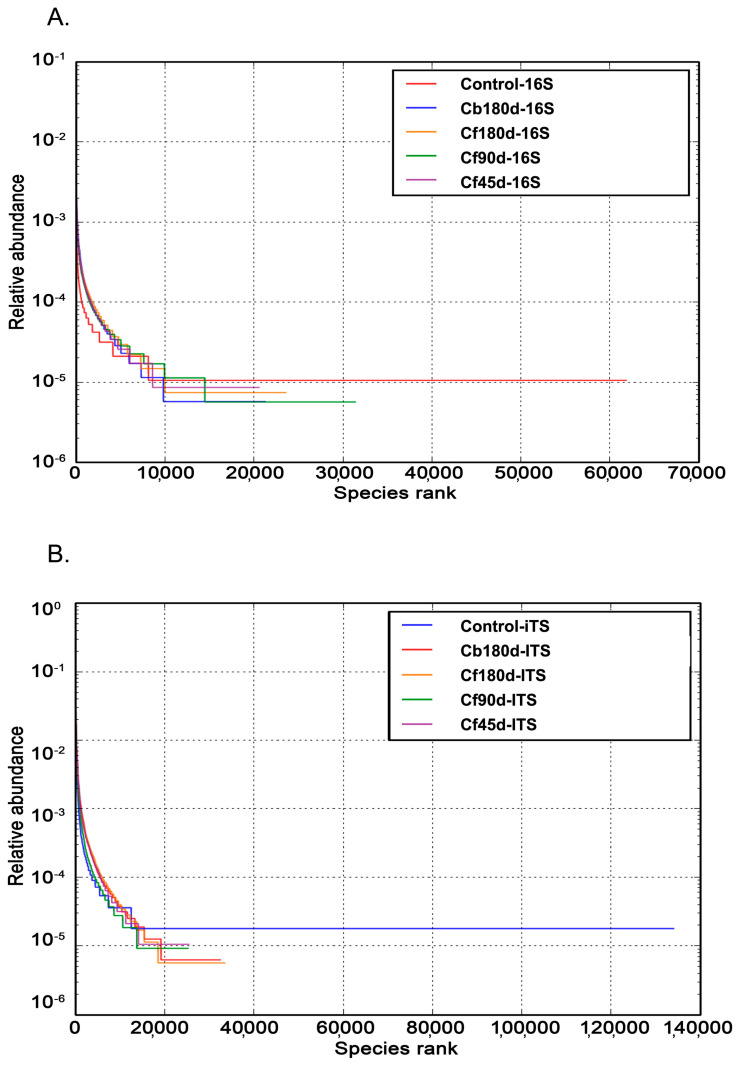
Rank abundance curve of (**A**) bacterial diversity (**B**) fungal diversity, at different developmental stages of *C. barbatus*.

The diversity of organisms present in a particular sample was summarized by alpha diversity. This is represented by a single number and was estimated from the distribution of the species-level annotations (Table 3). The beta diversity index is the diversity matrix used to represent the explicit comparison of microbial communities for each pair of samples, on the bases of their composition. Betadiversity metrics thus assess the differences between microbial communities. The fundamental output of these comparisons is a square matrix where a “distance” or dissimilarity is calculated between every pair of community samples, reflecting the dissimilarity between those samples. A higher number in the matrix represents more dissimilarity and zero indicates no dissimilarity between samples. The dissimilarity matrix of Control-16S, Cb180d-16S, Cf180d-16S, Cf90d-16S, and Cf45d-16S, as well as Control-ITS, Cb180d-ITS, Cf180d-ITS, Cf90d-ITS, and Cf45d-ITS, is shown in Table 4. The relative species abundance is represented by the rank abundance curve shown in Figure 8. The rank abundance plot can be used for visualizing the number of different species, i.e., species richness, and the closeness in the number of species, i.e., species evenness [58].

## 4. Discussion

Soil provides carbon sources, nitrogen sources, and growth factors for the growth of microorganisms present in it [59]. A favorable environment is responsible for richness in microbial diversity and alters soil and plant microbiomes [60]. The root exudes various water-soluble metabolites and volatile organic compounds that accumulate in the root, into the rhizosphere soil, which are capable of stimulating or inhibiting the growth of microbes in the rhizosphere. For instance, a classic example of plant–microbe interaction influenced by chemicals present or released from roots is when a wounded plant releases acetosyringone, which acts as a chemo-attractant for *Agrobacterium* and leads to crown gall disease in the plant [61,62]. In turn, rhizospheric microbes also benefit the plant in different ways or can also act as pathogens. The present study focuses on exigent issues, including how these rhizospheric microbes compete and interact with each other, as well as with the plant, for their survival, which can help provide an understanding of the microbial ecology of *C. barbatus*. Furthermore, the developmental stages of plants and animals including humans have an impact on the microbial diversity present in their ecosystem [9,29,30,63,64,65]. These can be understandable to a large extent through rhizosphere metagenomic study but it is challenging to link the metagenomic information with the organism or ecosystem from where the DNA was isolated.

Our aim during this study was to investigate the influence of plant growth stages on the microbial communities of rhizosphere in the medicinal plant *C. barbatus*. We hypothesized that the forskolin and other plant metabolites may have some impact on the rhizosphere microflora and vice versa. Results obtained from this study revealed that the alpha diversity of all samples was less than that of the control (Table 3). Moreover, the rhizosphere of *C. barbatus* was found to be enriched with bacterial diversity as compared to *C. blumei*. Interestingly, in the metagenomic study, the fungal diversity was lower in both *C. barbatus* and *C. blumei* compared to that of the control. This variation in the specific rhizosphere microbial communities could be due to the forskolin or related metabolites accumulated in the roots of *C. barbatus*. The main economic part of the *C. barbatus* is its tuberous roots and 90 d old plants produced significantly higher root yields and forskolin content. Thus, the accumulation levels of forskolin in *C. barbatus* positively correlate with the biomass of roots, as also supported by earlier reports [66,67,68].

In the present study, the most abundant bacterial phyla detected in the 16S metagenomic library were Proteobacteria, Acidobacteria, Actinobacteria, Bacteroidetes, and Planctomycetota. Abundance of Proteobacteria and Acidobacteria was earlier reported in the rhizosphere of different varieties of *Zea mays* [69,70], *Triticum aestivum* [71], *Rumexpatientia* [72], and *Dendrobium* sp. [73]. Dominance of Proteobacteria, Acidobacteria, Actinobacteria, Bacteroidetes, and Fermicutes phyla was also reported in the rhizospheric microbiome of watermelon, cotton, rice, and *Arabidopsis* [74,75,76]. Some bacterial species of Proteobacteria phylum were reported to produce siderophores and indole-3-acetic acid (IAA), as well as have a potential role in nitrogen fixation and show inhibitory activity against phytopathogens [77]. Various reports have documented that Actinobacteria plays an important role in tolerance to abiotic and biotic stresses, drought tolerance, phosphorus utilization, and increasing plant vigor [78,79,80]. A study has shown that the phylum Chloroflexi was dominant in rhizosphere soil of *Ludwigiaprostrata* and *Fimbristylismiliacea* and it may have a role in cadmium (Cd) tolerance [53]. A study showed that members of *Kaistobacter* genus were detected in soil rich in isoprene [81]. Isoprene units are the building block of terpenes and forskolin is also a diterpene. Thus, it may be possible that *Kaistobacter* was attracted towards the rhizosphere of *C. barbatus* because of these isoprene units. Moreover, in our study through forskolin quantification, we found that root extracts prepared from three-month-old plants hadthe highest forskolin content compared to six-month-old and 45 d old plants, which resembled the abundance pattern of *Kaistobacter* (Figure 5).

As shown in Figure 6, we found the highest percentage abundance of 43.50% of Ascomycota in the control sample, followed by Cb180d (38.50%), and a lower abundance in Cf180d (19.00%), Cf90d (20.70%),and Cf45d (22.90%). Our results are comparable with previous studies where Ascomycota was reported as the dominant phylum, representing 33.18% of the OTUs in *Salix repens* followed by Basidiomycota [82]. Recently, Ascomycota and Basidiomycota were reported as a dominant fungal phylum in the rhizosphere of *Larixdeciduas* [83]. The microbiome of *C. barbatus* showed that the Agaricomycetes, Sordariomycetes, Dothideomycetes, Eurotiomycetes, Exobasidiomycetes, and Chytridiomycetes classes were dominant in all samples. Similar results have been reported from the rhizosphere of *Panax notoginseng*, wherein the phylum Ascomycota, Zygomycota, Basidiomycota, and Chytridiomycota were found to be dominant [84]. A member of the class Dothideomycetes is *Phoma* sp., which contains several plant pathogenic species [85]. Interestingly, in our study we found the genus *Phoma* to have a significantly low abundance in *C. barbatus* compared to *C. blumei*. Moreover, *Phomaherbarum* was reported to cause root rot disease in *Panax notoginseng* [86]. Thus, it may be possible that the growth of *Phoma* sp. is inhibited because of the accumulation of forskolin or any other metabolite in the roots of *C. barbatus*.

Several reports have showed that plant secondary metabolites leach out into the rhizosphere soil as root exudates [87,88,89]. Terpenes, flavonoids, glucosinolates, and alkaloids are present in root exudates and function as defensive or signaling compounds. Apart from direct exudation, metabolites may also be released into rhizosphere soil through plant litter and decomposition of root tissues [90]. For instance, benzoxazinoids, which include benzoxazinones and benzoxazolinones, are unique bioactive metabolites produced by certain members of the Poaceae, including maize, wheat, rye, and some dicots. Metabolite profiling showed the presence of benzoxazinoids and their derivatives in rhizosphere soil [88]. Terpenoid derivatives were found in the root exudates of maize plants, while their quantity in exudates increased on treatment with endophytic diazotroph *Herbaspirillum seropedicae*, exemplifying the impact of microflora on rhizospheric metabolites [91]. Allelopathic diterpenes were found to be present in the soil samples where *Cistus ladanifer* was growing [92]. Monoterpene 1,8-cineole was identified as a rhizosphere volatile released by *Arabidopsis thaliana* [93]. Similarly, terpenes, produced by Lamiaceae species *Salvia leucophylla* and *Thymus vulgaris*, have been found in the rhizosphere soils of these plants [94,95]. It is likely that the secondary metabolites of *C. barbatus* may also be released into the rhizosphere soil, either directly or through root disintegration, and may impact the rhizosphere ecology.

## 5. Conclusions

In this study, bacterial and fungal diversity were studied in the rhizosphere of *C. barbatus* at different developmental stages and also compared with rhizosphere microflora of *C.blumei*. The age and growth stages of *C. barbatus* are associated with the community structure of rhizosphere microflora. The *Kastiobacter* genus was abundant in the rhizosphere of *C. barbatus* during the developmental stage when it is most metabolically active. Representation of the *Phoma* genus was lower in the rhizosphere of *C. barbatus* compared with that of *C. blumei*. Remarkable differences in metabolites levels, including forskolin and the rhizosphere microbes, were observed during different developmental stages in *C. barbatus*. This will help us to explore the ecological role of metabolites, particularly forskolin. This knowledge will be also vital for understanding how forskolin affects the microbial diversity of the rhizosphere, as well as how microbial diversity present in the rhizosphere affects *C. barbatus* during different developmental stages. Future studies should consider our work, in which we used *C. blumei* asa control (deprived of forskolin) when trying to understand the importance of plant developmental stages as well as the impacts of forskolin stored in the roots on rhizosphere diversity. Collectively, results obtained from metagenomic analysis also strengthen the argument that the developmental stages of a particular plant impose a greater impact on the succession of bacterial and fungal communities.

## Figures and Tables

**Table 1:** Sequences of primers used in study.

	Primer Name	Primer Sequence (5′ to 3′)	Length of Primer
16S	V3-Forward	CCTACGGGNGGCWGCAG	17
	V4-Reverse	GACTACHVGGGTATCTAATCC	21
ITS	ITS2-Forward	GCATCGATGAAGAACGCAGC	20
	ITS2-Reverse	TCCTCCGCTTATTGATATGC	20

**Table 2:** Read statistics of all samples.

Sample Name	No. of Reads
Control-16S	229,680
Cb180d-16S	350,216
Cf180d-16S	362,838
Cf90d-16S	352,793
Cf45d-16S	323,549
Control-ITS	253,061
Cb180d-ITS	106,639
Cf180d-ITS	196,431
Cf90d-ITS	198,977
Cf45d-ITS	140,556

**Table 3:** α-Diversity of all samples.

Sample Name	Shannon Alpha Diversity	Observed Species
Control-16S	15.03	61,832
Cb180d-16S	11.82	21,231
Cf180d-16S	12.39	23,554
Cf90d-16S	12.60	31,371
Cf45d-16S	11.93	20,555
Control-ITS	8.57	13,396
Cb180d-ITS	7.23	2531
Cf180d-ITS	7.12	3241
Cf90d-ITS	7.04	3342
Cf45d-ITS	6.47	2516

**Table 4:** β-diversity of all samples.

	**Control-16S**	**Cb180d-16S**	**Cf180d-16S**	**Cf90d-16S**	**Cf45d-16S**
Control-16S	0	6322.79	3594.24	5601.30	4720.02
Cb180d-16S	6322.79	0	7289.51	8856.55	7668.94
Cf180d-16S	3594.24	7289.51	0	6092.86	5962.04
Cf90d-16S	5601.30	8856.55	6092.86	0	7949.65
Cf45d-16S	4720.02	7668.94	5962.04	7949.65	0
	**Control-ITS**	**Cb180d-ITS**	**Cf180d-ITS**	**Cf90d-ITS**	**Cf45d-ITS**
Control-ITS	0	14,457.61	33,958.67	29,702.54	23,094.09
Cb180d-ITS	14,457.61	0	33,013.18	32,609.14	24,629.59
Cf180d-ITS	33,958.67	33,013.18	0	43,308.69	38,896.66
Cf90d-ITS	29,702.54	32,609.14	43,308.69	0	34,828.69
Cf45d-ITS	23,094.09	24,629.59	38,896.66	34,828.69	0

## Data Availability

Not applicable.

## Supplementary Materials

The following supporting information can be downloaded at: https://www.mdpi.com/article/10.3390/microorganisms11030705/s1, Figure S1: Bacterial diversity in rhizosphere soil at different developmental stages of *C. barbatus* at class level; Figure S2: Bacterial diversity in rhizosphere soil at different developmental stages of *C. barbatus* at order level; Figure S3: Bacterial diversity in rhizosphere soil at different developmental stages of *C. barbatus* at family level; Figure S4: Fungal diversity in rhizosphere soil at different developmental stages of *C. barbatus* at class level; Figure S5: Fungal diversity in rhizosphere soil at different developmental stages of *C. barbatus* at order level; Figure S6: Fungal diversity in rhizosphere soil at different developmental stages of *C. barbatus* at family level.
