# Supplementary figures and images for: Changing Rhizosphere Microbial Community and Metabolites with Developmental Stages of Coleus barbatus
Source: Microorganisms. 2023 Mar 9;11(3):705. doi: 10.3390/microorganisms11030705 (PMC10056624; doi:10.3390/microorganisms11030705)

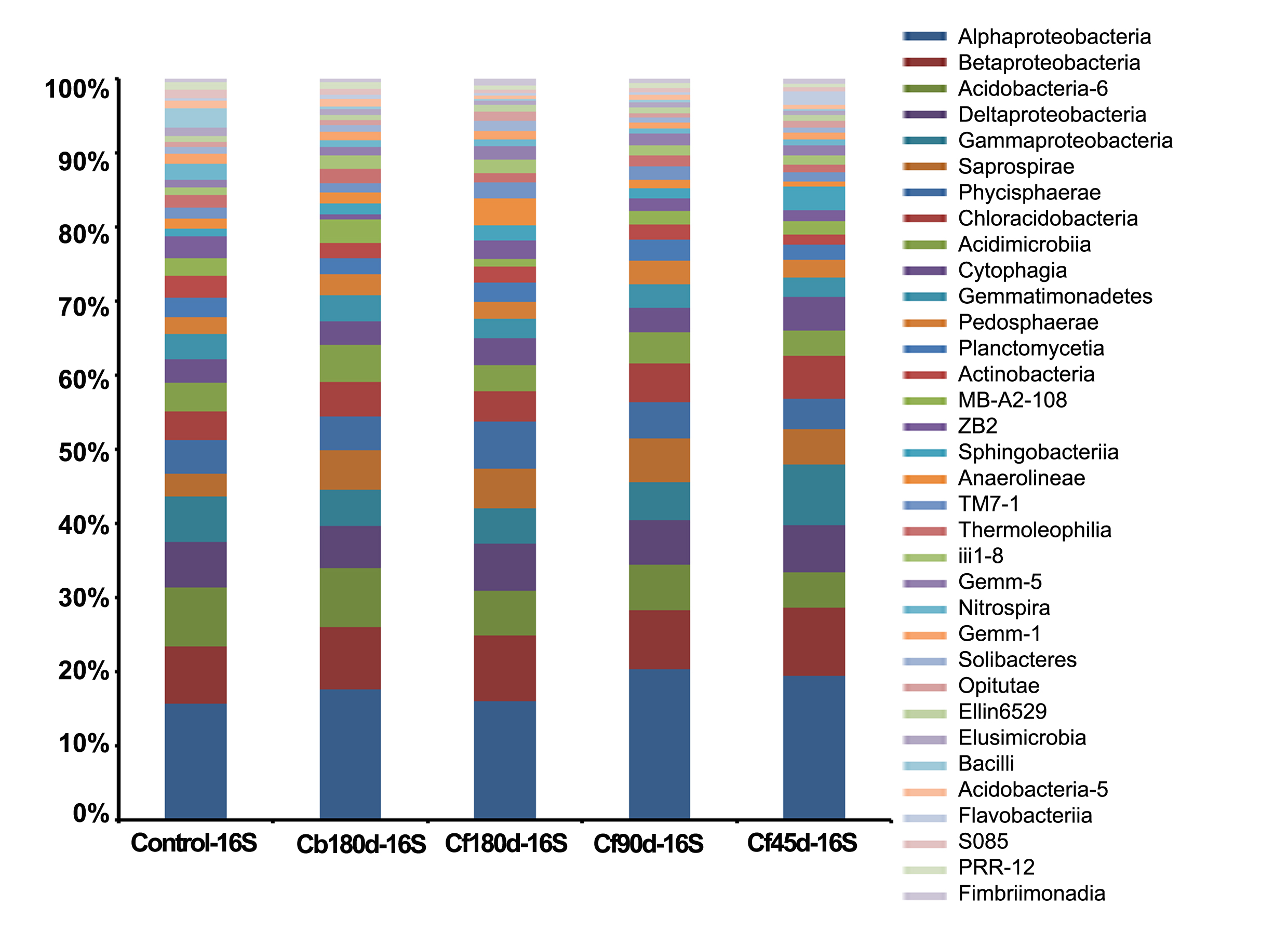

Supplement: Supplementary file 1 [file microorganisms-11-00705-s001.zip › Supplementary Figure S1.jpg]

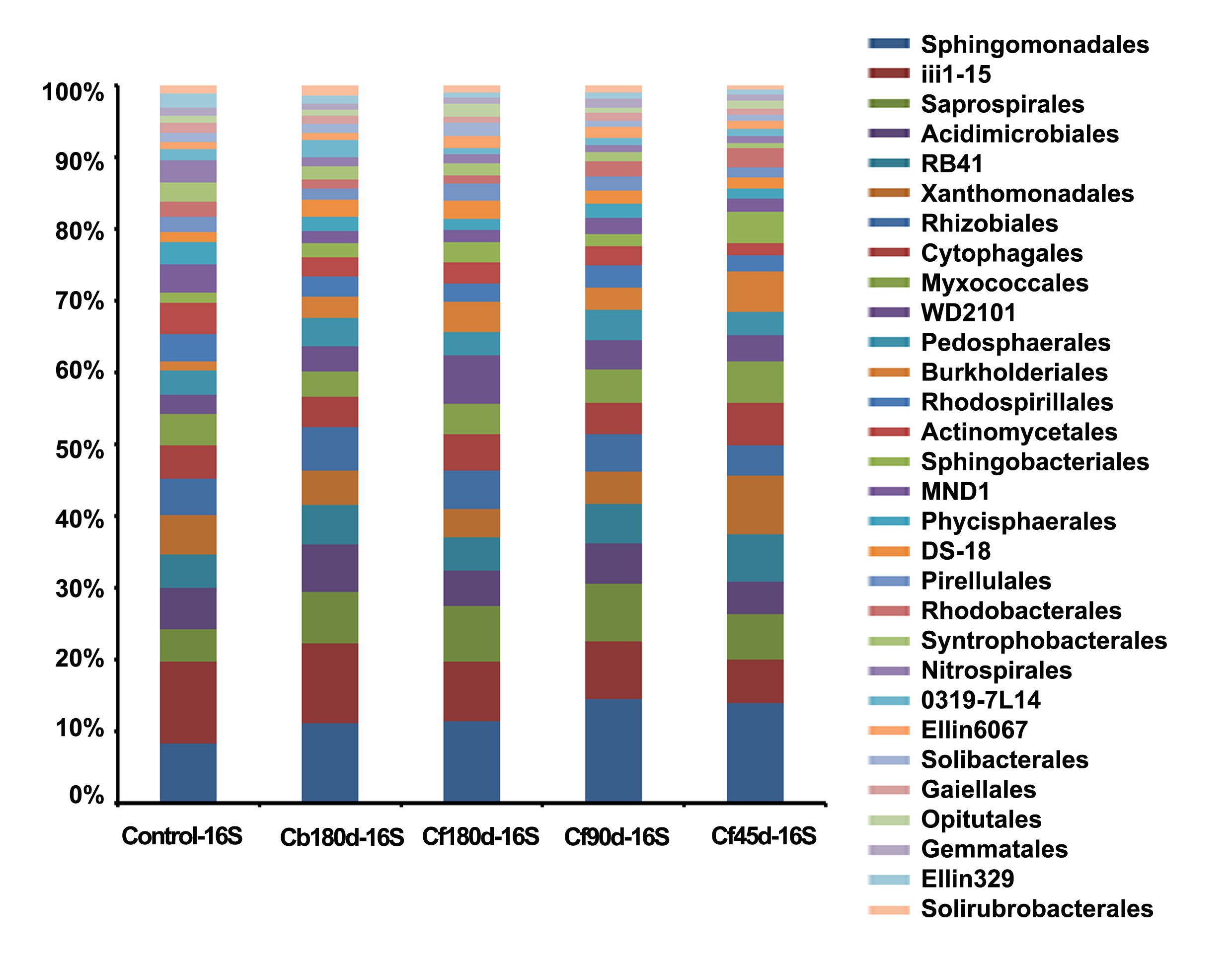

Supplement: Supplementary file 1 [file microorganisms-11-00705-s001.zip › Supplementary Figure S2.jpg]

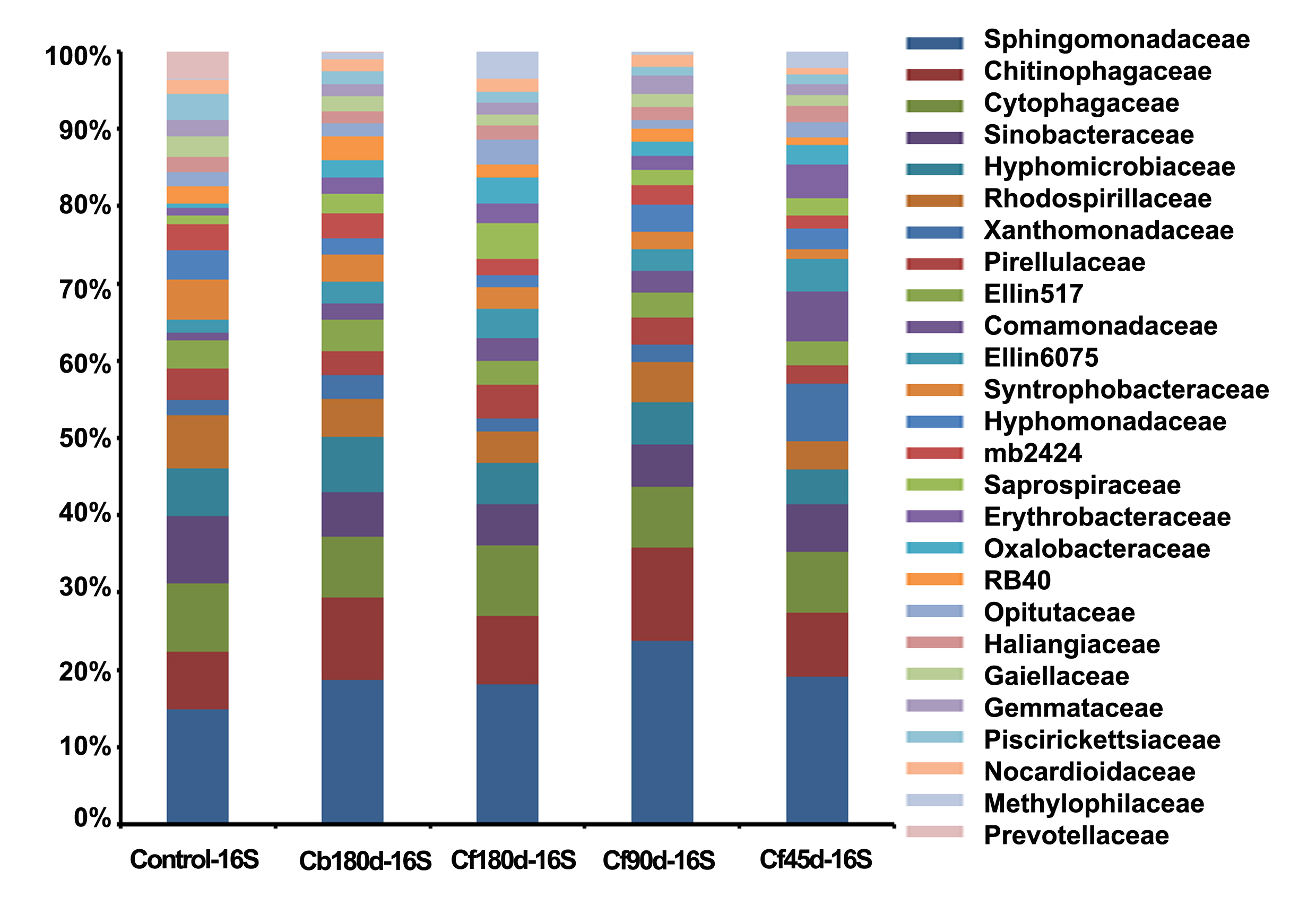

Supplement: Supplementary file 1 [file microorganisms-11-00705-s001.zip › Supplementary Figure S3.jpg]

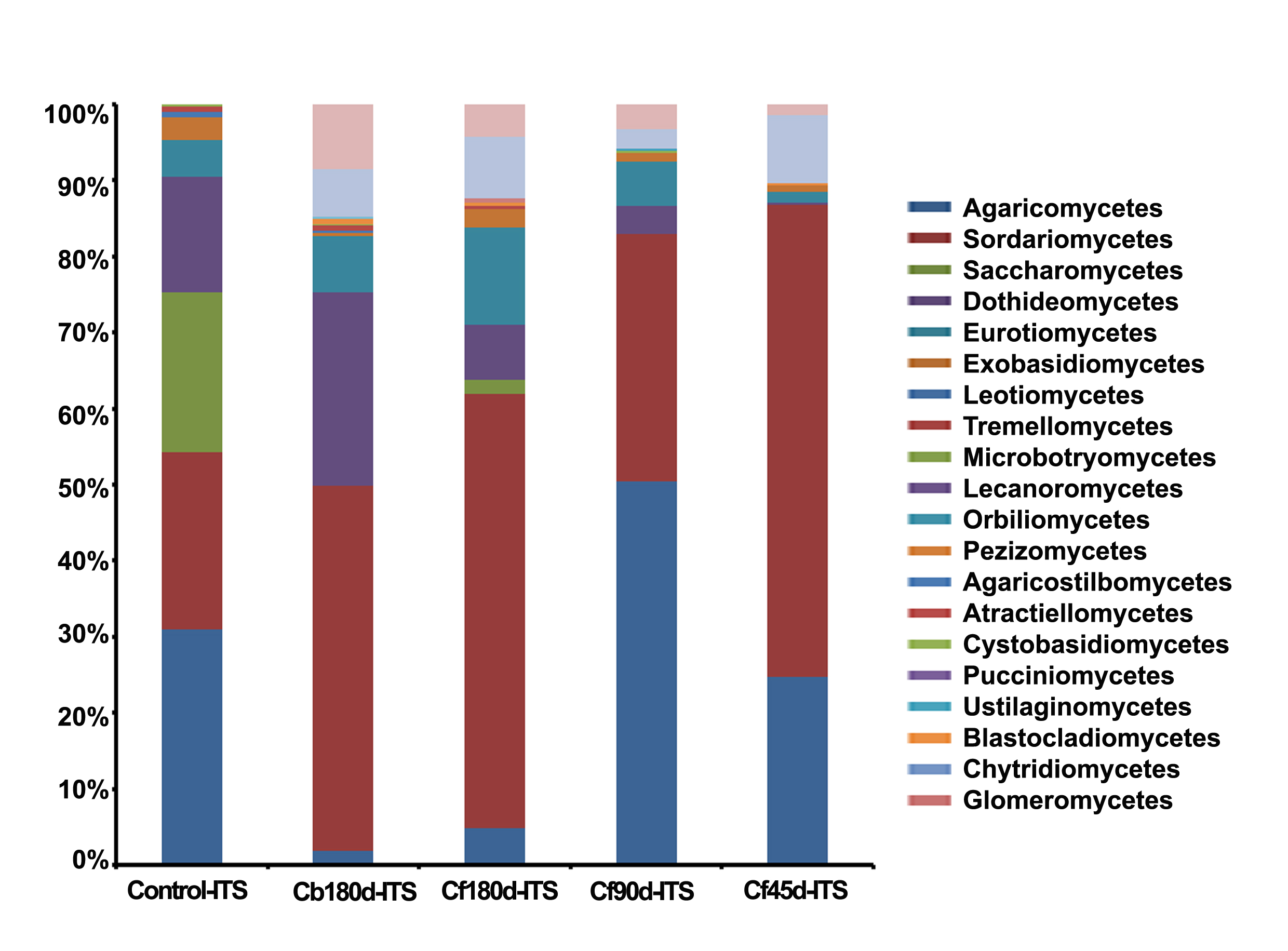

Supplement: Supplementary file 1 [file microorganisms-11-00705-s001.zip › Supplementary Figure S4.jpg]

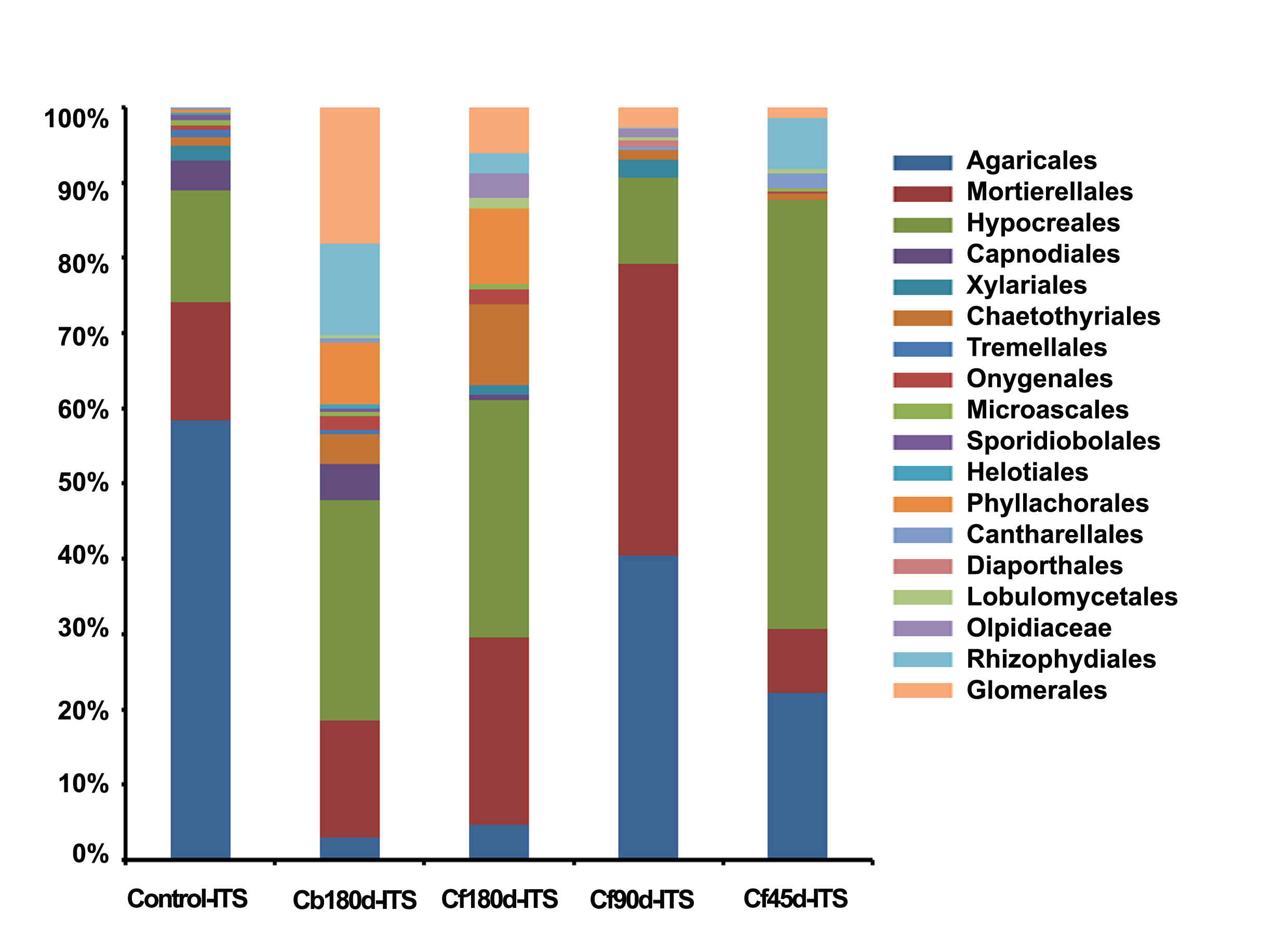

Supplement: Supplementary file 1 [file microorganisms-11-00705-s001.zip › Supplementary Figure S5.jpg]

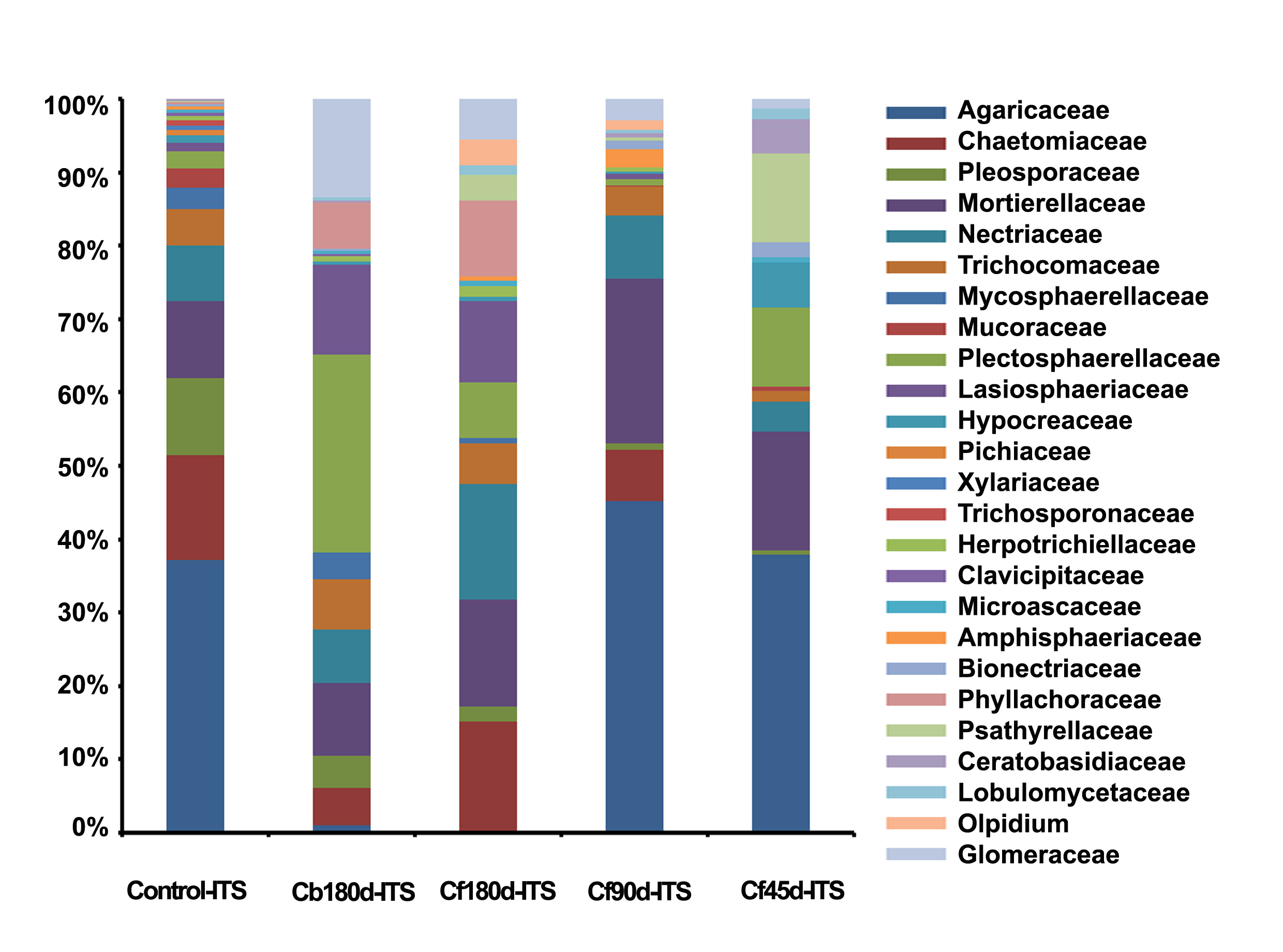

Supplement: Supplementary file 1 [file microorganisms-11-00705-s001.zip › Supplementary Figure S6.jpg]
